# ROSE-X: an annotated data set for evaluation of 3D plant organ segmentation methods

**DOI:** 10.1186/s13007-020-00573-w

**Published:** 2020-03-04

**Authors:** Helin Dutagaci, Pejman Rasti, Gilles Galopin, David Rousseau

**Affiliations:** 1 LARIS, UMR INRA IRHS, Université d’Angers, 62 Avenue Notre Dame du Lac, 49000 Angers, France; 2 INRA, UMR1345 Institut de Recherche en Horticulture et Semences, 42 Georges Morel CS 60057, 49071 Beaucouze, France; 3 ESAIP, école d’ingénieur informatique et environnement, Saint Barthélemy d’Anjou, France

**Keywords:** X-ray, Rosebush, Segmentation, Database, Machine learning

## Abstract

**Background:**

The production and availability of annotated data sets are indispensable for training and evaluation of automatic phenotyping methods. The need for complete 3D models of real plants with organ-level labeling is even more pronounced due to the advances in 3D vision-based phenotyping techniques and the difficulty of full annotation of the intricate 3D plant structure.

**Results:**

We introduce the ROSE-X data set of 11 annotated 3D models of real rosebush plants acquired through X-ray tomography and presented both in volumetric form and as point clouds. The annotation is performed manually to provide ground truth data in the form of organ labels for the voxels corresponding to the plant shoot. This data set is constructed to serve both as training data for supervised learning methods performing organ-level segmentation and as a benchmark to evaluate their performance. The rosebush models in the data set are of high quality and complex architecture with organs frequently touching each other posing a challenge for the current plant organ segmentation methods. We report leaf/stem segmentation results obtained using four baseline methods. The best performance is achieved by the volumetric approach where local features are trained with a random forest classifier, giving Intersection of Union (IoU) values of 97.93% and 86.23% for leaf and stem classes, respectively.

**Conclusion:**

We provided an annotated 3D data set of 11 rosebush plants for training and evaluation of organ segmentation methods. We also reported leaf/stem segmentation results of baseline methods, which are open to improvement. The data set, together with the baseline results, has the potential of becoming a significant resource for future studies on automatic plant phenotyping.

## Background

Recent agricultural and genetic technologies require high throughput phenotyping systems which can benefit significantly from the automation of inspection and measurement. Automatic plant phenotyping through 3D data has been a recent research topic in computer vision; however, the scarcity of labeled and complete models of real plants is a roadblock for applying recent machine learning techniques that rely on a vast amount of annotated data. Also, benchmarking data sets are indispensable for proper comparison of current and future phenotyping methods that operate on 3D data such as volumetric models or point clouds.

The production of annotated data sets has become even more important since the recent bloom of deep learning [1], performance of which was shown to be notably boosted by the availability of large annotated data sets [2]. The success of deep learning methods has triggered the interest in data collection and labeling in specific applications of computer vision such as plant imaging [3]. Most of the freely available annotated plant shoot data sets so far have been in the form of collections of 2D images acquired in the visible spectrum from top or side view. Among the available 2D data sets reported in [3] some are provided with annotated ground truth [4, 5], which is very valuable for phenotyping through computer vision and machine learning. In this article, we are interested in providing 3D annotated models of plants.

Among the most related data sets, some provide multiple images of plants that would allow 3D reconstruction; however, they do not include complete 3D plant models [6–9]. Uchiyama et al. [7] provided a data set containing multiple RGB and depth images of Komatsuna plant together with the manually annotated leaf labels. The data set contains calibration images to be used for estimating 3D geometry from the plant images. Cruz et al. [8] constructed a database named “MSU-PID” containing fluorescence, IR, RGB, and top view depth images of Arabidopsis and bean plants. 3D reconstructions of plants are not available in the database. Bernotas et al. [9] provided an annotated Arabidopsis data set with 3D information acquired using the photometric stereo technique. The data set includes 221 manually annotated Arabidopsis rosettes, which are partially reconstructed using only top-down views of the plants, providing 2.5D information rather than full 3D models. Wen at al. [10] introduced a database of the 3D models of plants and organs from different species, cultivars, and multiple growth periods, however, at present, the majority of the models in the data set correspond to isolated organs, such as models of single leaves or fruits, rather than full plants.

Due to the improvement of the sensitivity of the sensors and the democratization of the technology, X-ray Computer Tomography (CT) is now widely used for plant imaging [11]. While X-ray imaging is the most adopted tool to monitor roots in real soil conditions [12], it is also being employed for the characterization of the aerial parts of plants [13–19]. The use of X-ray imaging has focused on the acquisition of very thin parts enhanced with dye [13, 17, 18] or the internal 3D analysis of the aerial part [14–16, 19].

Rosebushes have been studied with computer vision techniques applied on LiDAR and RGB image data [20, 21] to produce global characterization of the shoot and from there estimate its ornamental value. In contrast to these optics-based methods, X-ray CT imaging, although more expensive, provides complete and occlusion-free volumetric information of the 3D geometric structure of the shoot. Such accurate imaging that is able to capture internal structures provides a means to construct full 3D models of real plants. These models can later be used to guide computer vision and pattern recognition techniques that can operate on data acquired with low-cost imaging devices to inspect a large number of plants used in typical phenotyping experiments.

We provide the ROSE-X data set of 11 complete 3D models of real potted rosebush plants with complex architecture acquired through X-ray computed tomography. The rosebushes we captured through X-ray CT imaging have complex architecture and show significantly high amounts of self-occlusion from all viewpoints, i.e., they possess major challenges for optics-based 3D plant reconstruction methods. These models are suitable to be transformed to other data structures, e.g., full or partial point clouds corresponding to the visible surface of the shoot, similar to what would be obtained with optical systems used for 3D reconstruction of plant shoot such as LiDAR or Time-of-Flight (ToF) cameras [22]. This conversion will make it possible to train and evaluate algorithms that operate on point clouds originating from the visible surface. In addition, with the data available for the occluded parts, these models will make it possible to design algorithms that predict complex plant architectural structure from incomplete input.

The 3D voxel space of each rosebush in the data set is fully annotated through labeling each voxel with its corresponding botanical organ class; “organ” referring to the plant units such as leaves, branches, and flowers. Such ground truth data facilitate the detailed description of the architecture and morphology of the plant, and can be used to train automatic phenotyping algorithms aiming to extract both architectural and organ-level traits. Architectural and organ-level trait analysis of 3D data requires an initial stage of classification of points into their respective categories. Current practice is to segment the acquired data of the plant shoot into branches and leaves. In this paper, we focus on leaf-stem segmentation algorithms as one of the phenotyping applications where our data set can serve both as training data and as a benchmark. We chose four representative methods for stem-leaf segmentation: (1) unsupervised classification using local features from point clouds, (2) support vector machine (SVM) classification using local features from point clouds, (3) random forest-based classification of local features from volumetric data, and (4) 3D U-Net applied on volumetric data. The later two were not previously applied to 3D plant organ segmentation problem. We trained and evaluated the methods on the new ROSE-X data set, and provided baseline performance results.

## Methods

### The ROSE-X data set

We introduce an open repository of complete 3D models of real rosebush plants with ground truth annotations at organ-level. The acquisition was performed through a 3D Siemens X-ray imaging system with a voltage range of 10–450 kV, using a tungsten transmission target and a 280-mA current. For this study, the system was operated with an 80-kV voltage. The number of projections was 900, and each radiograph was an average of three exposures of 333 ms each to reduce the noise. The acquisition time per plant was 20 min. A total number of 11 rosebush plants with varying architectural complexity were imaged. The output data obtained from each acquisition session is a stack of X-ray images with a pixel spacing of 0.9766 mm and slice spacing of 0.5 mm. The data is represented in a 3D voxel space, where the intensity of each voxel reflected the material properties of the plant shoot at that voxel.

From the raw volume data, the 3D voxels belonging to the rosebushes and their pots were extracted through masking and thresholding. The masks were manually constructed to separate unrelated material coming from the imaging platform, and thresholding was performed to separate the plant voxels from the air. Table 1 gives the number of thresholded voxels, the number of voxels corresponding to the plant shoot, and the number of voxels on the surface of the plant shoot. The pot contains a significant portion of the voxels of the models; the large difference in the number of the voxels between models is due to different sizes of the pots. The plant shoot corresponds to the plant parts above the soil. Most of the voxels of the plant shoot are on the surface since leaves and petals and sepals of the flowers are very thin structures.

**Table 1:** Number of voxels in the models (also the number of points in the corresponding point cloud)

Model ID	# Thresholded voxels	# Plant shoot voxels	# Plant shoot surface voxels
S268650	794,618	312,212	275,954
S268660	588,101	157,029	127,158
S270230	657,195	205,686	175,800
S270240	642,192	169,276	142,474
S270250	818,568	347,013	301,786
S271780	2,091,739	305,534	264,634
S271790	2,072,313	200,346	171,963
S271800	2,011,882	164,108	138,065
S273080	1,153,337	176,155	145,284
S273090	1,909,986	192,755	166,246
S273110	1,254,316	294,528	257,992

**Fig. 1:**
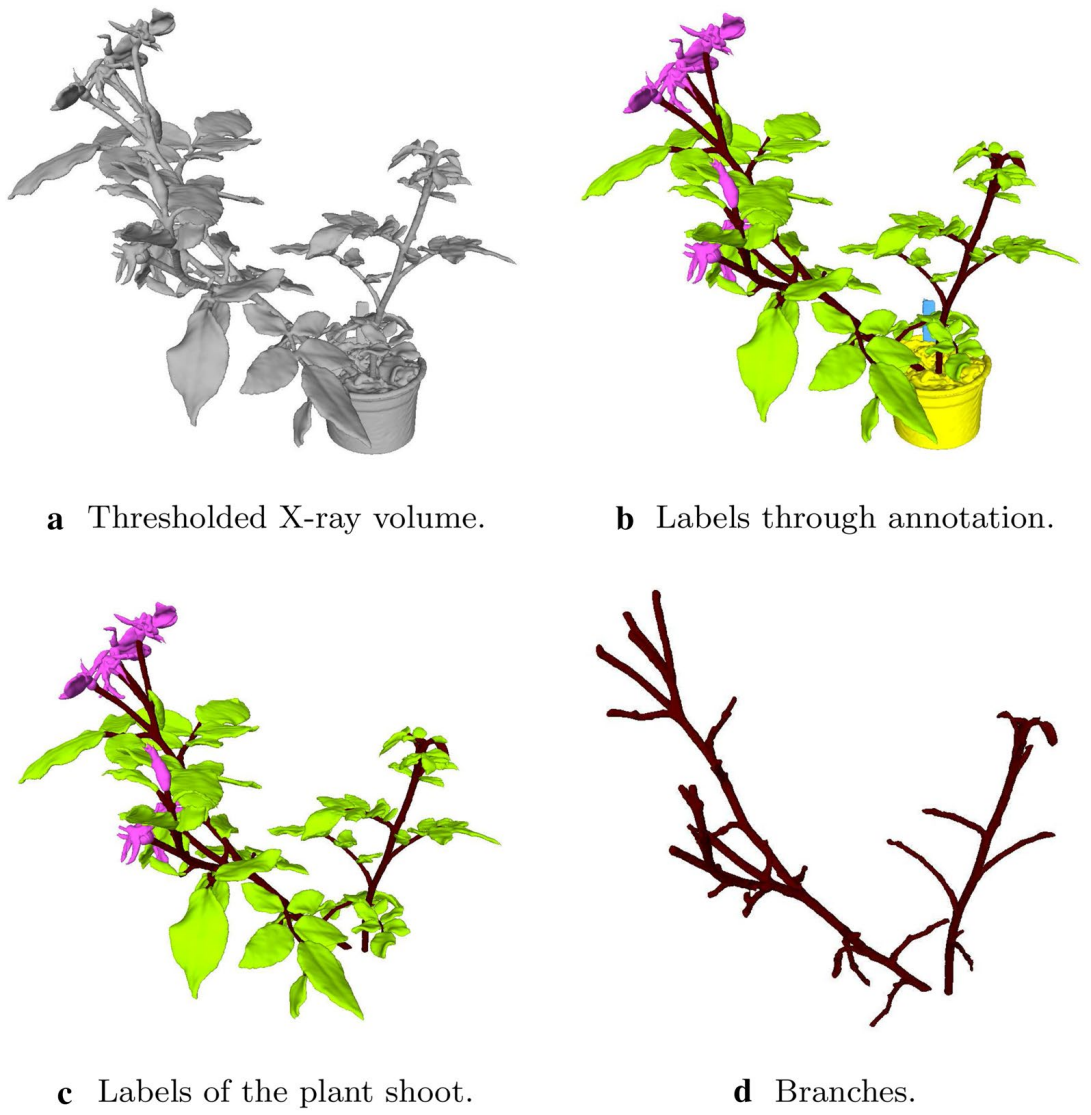
A sample rosebush model from the data set. The raw X-ray volume is thresholded and masked to obtain the solid part shown in **a**. Each voxel in the volume is annotated as leaf, stem, flower, pot, or tag to obtain the ground-truth segmentation as shown in **b**. In **c** only the parts corresponding to the plant shoot are shown, excluding the pot and the tag. The voxels corresponding only to stem class are shown in **d**

After the X-ray intensity values of the voxels corresponding to air and background material are set to zero, the remaining voxels are assigned to one of the following classes: (1) stem, (2) leaf, (3) flower, (4) pot, (5) tag. The background voxels corresponding to air were assigned “zero” values. The stem class includes both the main branches and the petioles since they have similar geometrical structures and are spatially connected. The plant shoot is composed of the stem, leaf, and flower classes. Figure 1 displays the thresholded X-ray volume (a), the organ-level labels obtained through annotation (b), the labels corresponding to the plant shoot (c), and the stem and petiole structure (d) of a sample rosebush model from the data set. Table 2 gives the percentages of voxels of organ classes on the plant shoot and the surface of the plant shoot.

**Table 2:** Percentages of voxels (points) for organ classes in the plant shoot

Model ID	Leaf	Stem	Flower	Leaf on surface	Stem on surface	Flower on surface
S268650	79.06	13.08	7.86	83.99	9.43	6.58
S268660	70.53	17.06	12.41	77.37	12.66	9.97
S270230	77.07	14.36	8.57	83.44	10.40	6.17
S270240	71.30	16.60	12.10	79.92	11.70	8.38
S270250	75.22	12.33	12.45	80.64	8.93	10.43
S271780	80.97	13.46	5.57	86.35	9.79	3.86
S271790	75.76	13.96	10.28	81.26	10.12	8.62
S271800	73.84	17.09	9.07	81.70	12.57	5.73
S273080	69.20	21.72	9.08	77.50	15.99	6.51
S273090	75.08	19.20	5.72	82.64	13.97	3.39
S273110	79.91	17.07	6.02	83.78	12.27	3.95

The manual annotation was carried out with the help of ilastik (Interactive Learning and Segmentation Toolkit) [23]: Using pixel classification tool of ilastik, on a rosebush model, we manually marked several voxels in regions belonging to each class to train the classifier. Then, we obtained full-volume predictions on all models generated by the trained classifier of ilastik. Through detailed inspection, we manually corrected the labels of all voxels incorrectly labeled by ilastik.

The data set is available online at [24]. We provide the 3D data in the following forms: (1) the raw X-ray image stack, (2) the binary volume mask indicating the voxels of only the shoot of the plant, the tag, and the pot, and the corresponding organ-level labels, (3) the binary volume mask indicating the voxels only on the surface of the plant shoot, and the corresponding organ-level labels, (4) the point cloud composed of the points of the shoot of the plant, the tag, and the pot with colors indicating organ-level labels, (5) the point cloud composed of the points on the surface of the plant shoot with colors indicating organ-level labels. The details of the file formats and label information are explained in the Additional file 1. Through these forms, it is possible to convert the 3D volumetric models to a labeled polygon mesh model and obtain 3D point clouds as viewed from any position around the plant through ray casting.

### Baseline methods for leaf-stem segmentation

Vision-based plant phenotyping has been traditionally performed through analysis of 2D color images from which 3D characteristics of the plants (stem length, volume, leaf area, etc.) have been estimated. With the advance of 3D imaging technologies, phenotyping through the 3D capture and reconstruction of plants have gained considerable attention. In Table 3, characteristics of some of the 3D vision-based phenotyping methods that involve a segmentation stage to separate leaves from branches are summarized. 3D data was captured from various species of plants by structured light depth sensors [25, 26], laser scanners [27–31], ToF cameras [32], or from a set of color images through structure from motion [33, 34].

**Table 3:** 3D vision based phenotyping methods

	Imaging	Plant type	Application/traits	Segmentation approach
*Local surface features on point clouds*				
Dey et al. [33]	Structure from motion	Grapevine	Classification of 3D points into leaves, branches, and fruit (red)	Eigenvalues of local covariance matrix, SVM, CRF
Li et al. [25]	Structured light scanner	Anthurium, Dishlia, Dancing bean	Leaf/stem segmentation for tracking events in time like budding and bifurcation	Local point features, MRF
Paulus et al. [28]	3D laser scanner	Grapevine, Wheat	Leaf/stem segmentation for grapevine	Local point features (FPFH), SVM, Region growing
Paulus et al. [35]	3D laser scanner	Barley	Leaf/stem segmentation for leaf area and stem height estimation	Local point features (FPFH), SVM, Region growing
Wahabzada et al. [30]	3D laser scanner	Grapevine, Wheat, Barley	Segmentation of leaf, stem, ear, and fruit parts	Local point features (FPFH), clustering, Region growing
Sodhi et al. [26]	Multi-view stereo & Kinect	Sorghum	Leaf/stem segmentation	Local point features (FPFH), SVM, CRF
Elnashef et al. [36]	Multi-view stereo	Corn, Cotton, Wheat	Leaf/stem segmentation	Eigenvalues of the second tensor
*Local features on volumetric models*				
Klodt et al. [37]	Multi-view stereo	Barley	Leaf/stem segmentation for the estimation of volume and surface area of the plant and the number of leaves	Eigenvalues of the second-moments tensor
Goldbach et al. [38]	Shape-from-silhouette	Tomato seedling	Leaf/stem segmentation for leaf length, width and area estimation	Breath-first flood-fill algorithm with a 26-connected neighbourhood
*Spectral clustering*				
Hétroy-Wheeler et al. [39]	Laser scanner	Tree seedlings	Segmentation of stems, leaves, and petioles for leaf surface area estimation	Graph construction, spectral embedding and clustering
Santos et al. [40]	Structure from motion	Sunflower, soybean	Leaf/stem segmentation for leaf surface area estimation	Graph construction, spectral embedding and clustering
*Geometric primitives*				
Binney and Sukhatme [31]	2D laser scanner	Tree branch	Segmentation of leaves and branches for estimation of branch locations, angles, radii, and lengths, and connectivity information between branches	Generative models for branches and branchpoints
Paproki et al. [41]	Multi-view stereo	Cotton	Stem, petiole and leaf segmentation for estimation of geometric properties such as stem height, leaf height and inclination angle	Region growing, tubular shape-fitting, clustering
Chaivivatrakul et al. [32]	Time of Flight	Corn	Leaf/stem segmentation for stem diameter, leaf length, area, and angle estimation	Stem extraction by ellipse fitting and linking, and elliptical cylinder extrusion
Gélard et al. [34]	Structure from motion	Sunflower	Stem, petiole and leaf segmentation for leaf area estimation	Ring climbing for extraction of stems and petioles, clustering for segmenting leaves

One of the disadvantages of these optics-based acquisition techniques is that they suffer from a high degree of self-occlusion of plants. As the architecture becomes more complex, more parts of the plants become heavily occluded, making it impossible to capture some regions from any viewpoint. That disadvantage forced most automatic part segmentation and phenotyping research to be conducted on plants with relatively simple architectural and geometrical structure, such as plants with a single stem and well-separated leaves. With X-ray imaging, 3D information of the entire plant material can be captured. However, many phenotyping activities, such as growth monitoring, require the plants not to be moved, which makes X-ray imaging impractical. The bulk of the automatic phenotyping activities is bound to rely on optics-based acquisition devices. Although X-ray imaging will remain as an appropriate tool for applications such as root growth analysis, we envision that the ROSE-X data set will be mainly a resource for algorithms that operate on point clouds acquired with optics-based methods. The availability of complete models of real plants with high architectural complexity and full annotation will serve as a guiding resource for processing occluded point clouds of highly complicated plants acquired by RGB or depth cameras, or laser scanners.

Whether the data is in 3D volumetric form or is in the form of a 3D point cloud, semantic segmentation is required for particular phenotyping objectives, such as organ-level phenotyping, extraction of the architecture and event detection such as leaf growth and decay. Leaf-stem segmentation is the most commonly addressed problem in organ-level phenotyping. We can categorize leaf/stem segmentation methods for 3D phenotyping into the following groups: (1) segmentation using local surface features on point clouds [25, 26, 28, 30, 33, 35], (2) segmentation using local features on volumetric data [37, 38], (3) segmentation through spectral clustering [39, 40, 42], (4) segmentation by fitting geometric primitives [31, 32, 34, 41, 43]. Table 3 is organized using this categorization. In this work, instead of an exhaustive evaluation of all the available methods on our labeled data set, we selected four representative approaches as baseline methods for segmenting the shoot of the rosebush data into its branches and leaves. Two of these methods are based on local features extracted from the point cloud. The other two methods assume volumetric data as input, and have not been previously applied to the plant organ segmentation problem. For all methods, it is assumed that the plant shoot is already separated from the pot. In the following subsections, the baseline methods are described in detail.

#### Segmentation using local surface features on point clouds

One of the most common approaches to segment point clouds of plants is to use local features. Point neighborhoods on leaves and branches exhibit distinguishing distributions, which can be attributed to their sheet-like or line-like structures, respectively. One of the simplest approaches is to represent such characteristics by the eigenvalues of the covariance matrix of the neighborhood. Researchers have devised the use of more sophisticated point features such as Fast Point Feature Histograms (FPFH) ([28, 35]) that provide a rich description of the local structure around a point. In this work, we opted to use the simplest point neighborhood descriptors for the baseline methods. For more information on 3D local features, we refer to the book [44] of Laga et al.

For a point *x* in the point cloud, the neighborhood can be defined as the set $\mathcal {N_x} = \{ x_i : \Vert x - x_i \Vert <d\}$, where *d* is the radius of the neighborhood. The covariance matrix of the neighborhood is calculated as $C = \frac{1}{|\mathcal {N_x}| -1} \sum _{x_i \in \mathcal {N_x}} (x_i - {\bar{x}})(x_i - {\bar{x}})^T$, where ${\bar{x}}=\frac{1}{|{\mathcal {N}}_x|} \sum _{x_i \in \mathcal {N_x}} x_i$ is the mean of the points.

The relative magnitudes of the eigenvalues $\{ \lambda _1, \lambda _2, \lambda _3 \}$ of the covariance matrix with $\lambda _1 \le \lambda _2 \le \lambda _3$ can serve as local descriptors to discriminate leaf and stem points. For a thin flat structure, we expect $\lambda _1$ to be much smaller than both $\lambda _2$ and $\lambda _3$. We also expect $\lambda _2$ and $\lambda _3$ to be close to each other. For a line-like structure we have a predominantly large value of $\lambda _3$, with $\lambda _1$ and $\lambda _2$ being much smaller.

We used the eigenvalues of the local covariance matrix in two baseline stem/leaf segmentation methods. The first is an unsupervised method based on the Markov Random Fields (MRF) formulation given in [25]. The second is a supervised method where a classifier is trained with local features derived from the eigenvalues. This second approach aligns with the methods proposed in [26, 33].

Local features on point clouds—unsupervised (LFPC-u) : For this baseline method, we followed a simplified version of the stem/leaf classification method given in [25]. The eigenvalues are used to define local surface features on the point clouds and to search for a mapping $f_B$ from a point *x* to one of the two labels for leaf (*L*) and stem (*S*) categories. The point cloud can be organized in a graph where the points $x\in {\mathcal {X}}$ correspond to the nodes and pairs of locally connected points $(x_i,x_j )\in {\mathcal {E}}$ constitute the edges. In our implementation, a pair $(x_i,x_j )$ was considered to be an edge if the Euclidean distance between them is less than 1.4mm. The energy associated with a particular label mapping is defined as

1 $$\begin{aligned} E(f_B) =w_D \sum _{x\in {\mathcal {X}}} D_x(f_B(x)) + w_V \sum _{(x_i,x_j )\in {\mathcal {E}}} V(f_B(x_i), f_B(x_j))\,. \end{aligned}$$

The weight factors $w_D$ and $w_V$ determine a compromise between the class likelihoods of individual nodes and the coherence across the edges. $D_x(f_B(x))$ corresponds to the data term (the unary potential) which gives the cost of classifying a point *x* into a leaf or stem category. The term $V(f_B(x_i), f_B(x_j))$ gives the smoothness term (the pairwise potential) and is used to encourage labeling coherence between neighboring points. The energy function is minimized through min-cut algorithm [45] to obtain the optimum labels for the point cloud.

To determine the data and smoothness terms, an estimate of the curvature at point *x* is computed using the eigenvalues of the covariance matrix as $C(x)=\frac{\lambda _1}{\lambda _1+\lambda _2+\lambda _3}$. The range of the curvature values is [0, 1/3], and leaf points are expected to have lower curvature values than stem points. A flatness feature is defined as $R(x) = log(max(C(x)),c_\epsilon )$, where $c_\epsilon$ is set to 0.015. *R*(*x*) is in the range $[R_L,R_S]$ with $R_L = log(c_\epsilon )$ and $R_S = log(1/3)$. Then, the data term is calculated as

2 $$\begin{aligned} D_x(f_B(x)) = {\left\{ \begin{array}{ll} R(x)-R_L, &{} \text {if }\, f_B(x) =L.\\ R_S-R(x), &{} \text {if }\, f_B(x) =S. \end{array}\right. } \end{aligned}$$

The smoothness term also depends on the curvature *C*(*x*), which is used as a measure of the discontinuity of the surface. The pairwise potential is set to be inversely proportional to the curvature since a high curvature value indicates a discontinuity which can be considered as the boundary of a plant part. The smoothness term is defined as

3 $$\begin{aligned} V(f_B(x_i), f_B(x_j)) = {\left\{ \begin{array}{ll} max\left( \frac{1}{C(x_i)}, \frac{1}{C(x_j)} \right) , &{} \text {if }\, f_B(x_i) \ne f_B(x_j).\\ 0, &{} \text {if } \, f_B(x_i) = f_B(x_j). \end{array}\right. } \end{aligned}$$

Notice that this method is an unsupervised method in the sense that it does not require labeled training data to transform or organize features to boost their discriminating power. However, the weight factors $w_D$ and $w_V$ in Eq. (1) need to be fixed. Through experimentation on one rosebush reserved to train the methods, we found that $w_D = 0.9$ and $w_N = 0.1$ yielded the best results.

Local features on point clouds—supervised (LFPC-s): For the second baseline method, we selected to derive local features from the eigenvalues of the local covariance matrix, and used SVM as the classifier as in the work of Dey et al. ([33]). We defined the local features as follows:

4 $$\begin{aligned} F_1 =\frac{\lambda _1}{\sqrt{\lambda _2 \lambda _3}} \quad F_2 =\frac{\lambda _2}{ \lambda _3} \quad F_3 =\frac{\lambda _1}{\sqrt{\lambda _1 \lambda _2 \lambda _3}} \quad F_4 =\frac{\lambda _1}{\lambda _2} \quad \end{aligned}$$

The size of the neighborhood from which the eigenvalues are computed determines the scale at which the local structures will be analyzed. The stem and the petioles of the plant shoot have varying widths, likewise the leaves exhibit a large size variability. Instead of fixing the radius, we extracted the features $\{F_1, F_2, F_3, F_4\}$ at various scales and concatenate them into a single feature vector. In our tests, we used six scales, corresponding to neighborhoods of radii 2, 3, 4, 5, 6, and 7 mm. Using one of the rosebush models with ground truth labels, we trained a two-class linear SVM classifier.

#### Segmentation using local features on volumetric data (LFVD)

The point cloud data acquired from optic-based sensors such as RGB cameras or laser scanners can be converted to binary volumetric data using a 3D occupancy grid. The regular structure of 3D volume allows to apply standard filtering and feature extraction tools such as smoothing and estimation of first and second order derivatives. The software ilastik [23] can extract various types of features from 3D volume data: the color features correspond to the raw intensity values smoothed by a Gaussian filter. The edge features are the eigenvalues of the structure tensor, eigenvalues of the Hessian matrix, the gradient magnitude of the difference of Gaussians and Laplacian of Gaussian. The texture features correspond to eigenvalues of the structure tensor, eigenvalues of the Hessian matrix, and orientation features are the raw structure tensor and Hessian matrix entries. In our tests, the mentioned features are extracted from data smoothed by Gaussian filters with scales 0.7, 1.0, 1.6, 3.5, 5.0, and 10.0 mm.

The voxels of the original X-ray data possess intensity values which are determined by the intensity of the X-rays passing through the voxels and the material properties. X-ray intensity values in our models depend on the material properties of plant parts; e.g., leaves have very low intensity values compared to branches. In order to have comparable results between the volume-based and surface-based baseline methods, we used the binary volume mask, indicating the voxels of only the shoot of the plant. We further set the values of the voxels which are not on the surface of the plant-shoot, i.e., interior voxels, to zero, so that only the voxels on the surface of the plant-shoot will remain.

We employed ilastik [23] to extract intensity, edge, and texture features from one binary plant model and to train a random forest classifier [46] using the ground-truth labels. Once the classifier is trained on one model; it is tested on all the other models on the data set.

#### CNN on volume data (3D U-Net)

As a representative of deep learning methods, we selected 3D U-Net [47], which is originally proposed to provide dense volumetric segmentation maps for bio-medical images. It is an extension of the 2D U-net architecture developed by Ronneberger et al. [48]; all the 2D operations in the 2D u-net are replaced with their 3D counterparts. The input volume is first passed through an analysis path with four resolution layers, each of which is composed of two $3\times 3\times 3$ convolutions with Rectified Linear Units (reLU) and one $3\times 3\times 3$ max pooling operation. Max pooling corresponds to downsampling by using the maximum value from each of a cluster of neurons at the prior layer. Then a synthesis path is applied with four resolution layers each consisting of one $2\times 2\times 2$ upconvolution operator followed by two $2\times 2\times 2$ convolutions with reLU. The high-resolution features obtained at the analysis path are also provided to the synthesis path through shortcut connections between layers of equal resolution. The size of the input voxel grid to the network is $144 \times 144 \times 144$, and the output is a volumetric data of the same size giving the label of each voxel. The architecture graph can be found in [47]. For more information on deep learning and the definitions of the classical layers that constitute the basis of deep neural networks, we refer to the book [49] of Goodfellow et al.

As we did with the baseline method based on local volumetric features, we only used the thresholded voxels on the surface of the shoot, so the input is binary devoid of the intensity information. We used one rosebush model to train the network. We extracted 25 subvolumes of size $144 \times 144 \times 144$ from various locations of the full volume of the model such that each subvolume contained leaf and stem instances. 20 of the subvolumes were used for training and 5 of them were used for validation. For a test model, we regularly partitioned the volume to non-overlapping subvolumes and provided the subvolumes to the network as inputs to get the corresponding segmentation.

## Results

In this paper, we concentrated on the problem of partitioning the plant models into its leaf and stem (branch) parts; so the training and evaluation of the baseline methods are performed using the ground truth labels corresponding to the leaves and stems only. In our evaluation, we ignored the predictions generated on the flower parts.

There are many metrics for segmentation evaluation, such as Matthews Correlation Coefficient [50], Cohen’s $\kappa$ coefficient [51], Dice Similarity Coefficient [52], all with their advantages and all applicable in the framework of our benchmark. In this paper, we used precision (also known as Positive Predictive Value), recall (also known as sensitivity), and Intersection over Union (IoU) to evaluate the baseline methods. Recall for the leaf class ($R_{leaf}$) is the ratio of the number of correctly labeled leaves (true positives) to the total number of leaf points in the ground truth (true positives + false negatives). Precision for the leaf class ($P_{leaf}$) is the ratio of the number of correctly labeled leaves (true positives) to the total number of points classified as leaf points by the algorithm (true positives + false positives). Recall ($R_{stem}$) and precision ($P_{stem}$) for the stem class are defined in the same way. Intersection over Union (IoU) metric for each class (IoU$_{leaf}$ and IoU$_{stem}$) is defined as the ratio of all the true positives to the sum of true positives, false negatives and false positives.

**Fig. 2:**
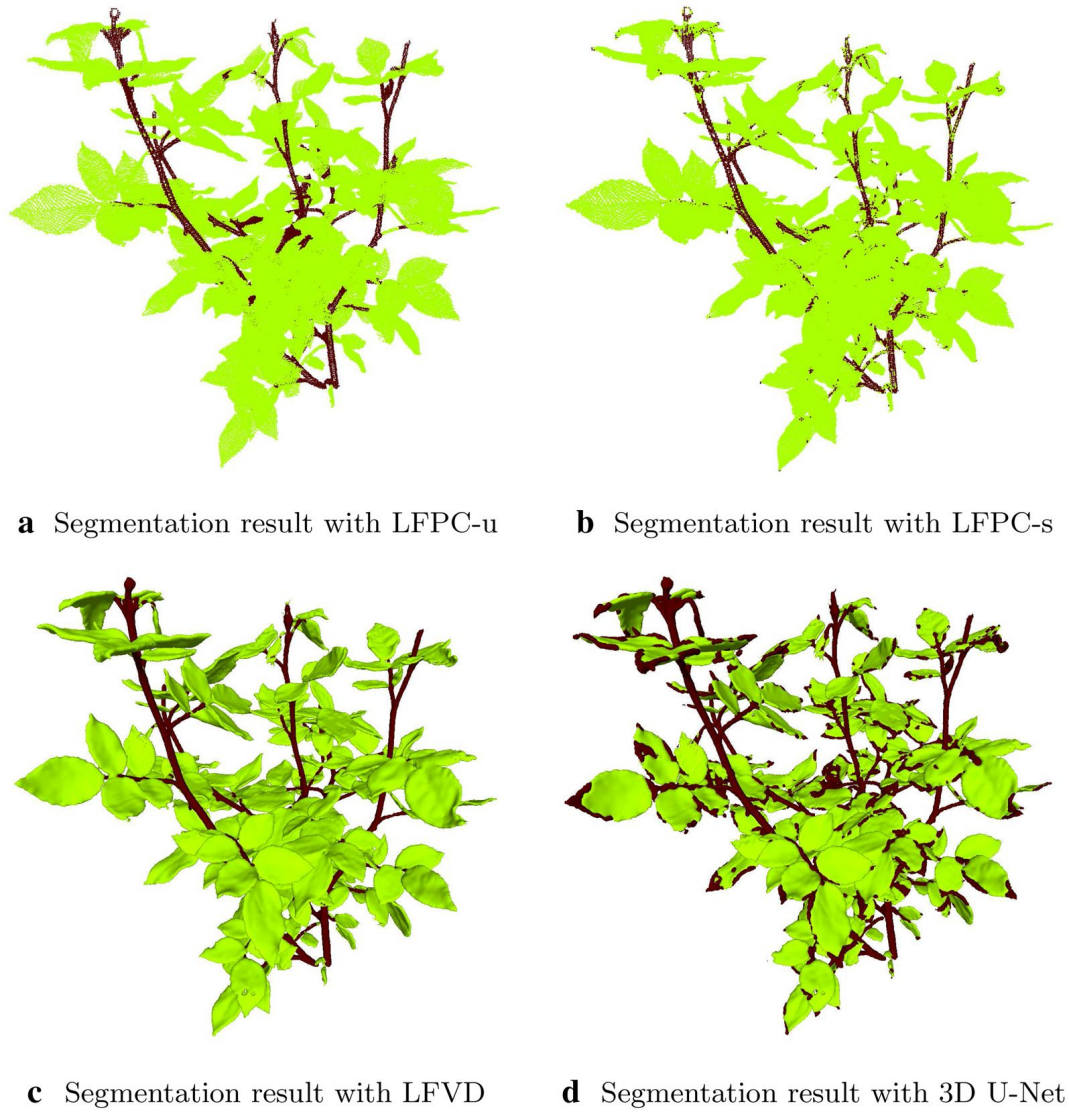
Leaf and stem labels predicted by the baseline methods for a sample test rosebush. The rendering is in volumetric form for LFVD and 3D U-Net and in point cloud for LFPC-u and LFPC-s. The methods LFPC-u (**a**) and LFVD (**c**) produced smooth results, while the labels predicted by LFPC-s (**b**) are slightly noisy. 3D U-Net (**d**) wrongly classifies leaf borders as stems

**Fig. 3:**
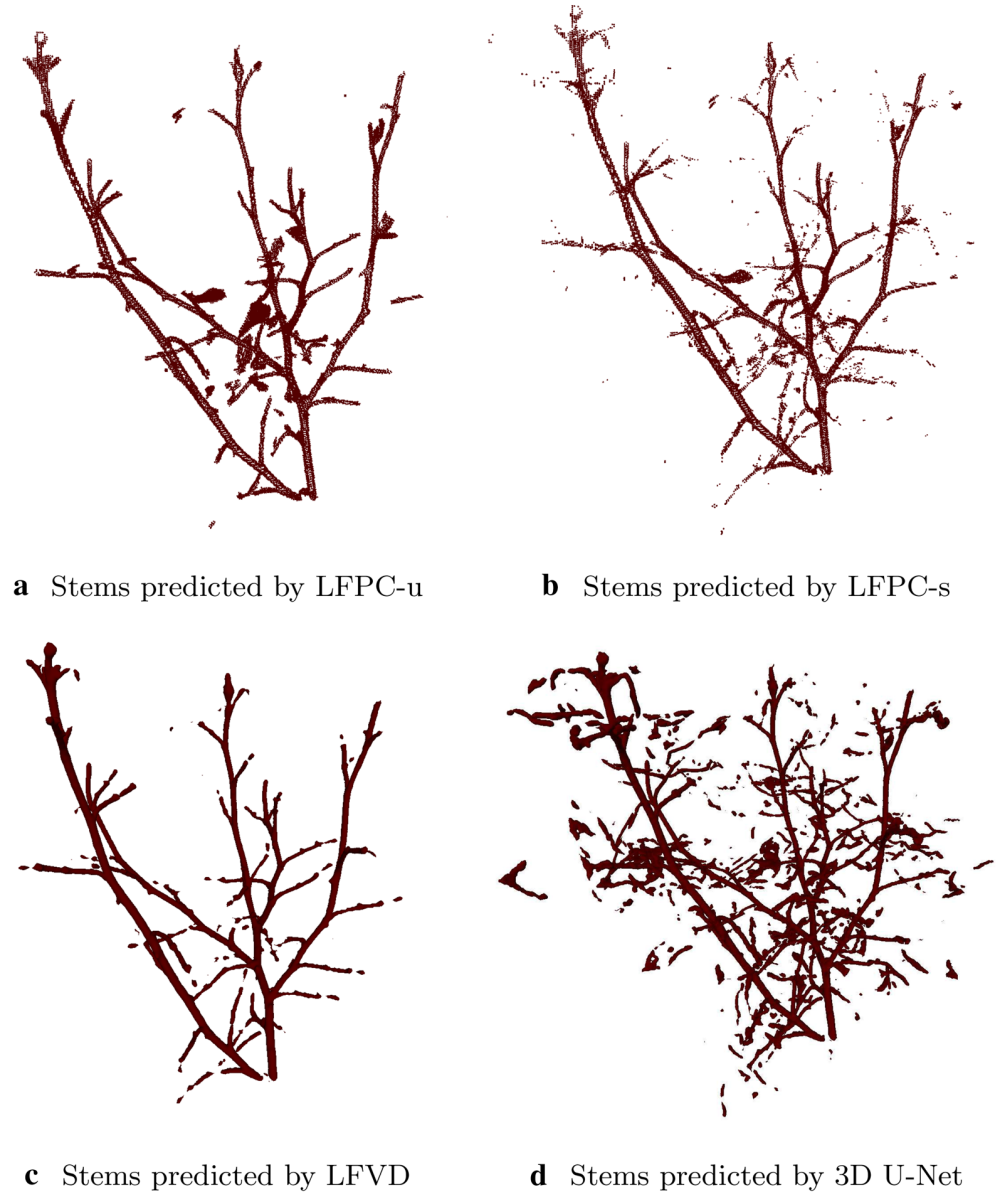
Stem labels predicted by the baseline methods for a sample test rosebush. The rendering is in volumetric form for LFVD and 3D U-Net and in point cloud for LFPC-u and LFPC-s. With the methods LFPC-u (**a**) and LFVD (**c**) the predicted stem structure is mostly connected, while LFVD (**c**) misses some petiole portions. The noisy predictions produced by the method LFPC-s (**b**) are more visible here. 3D U-Net (**d**) classifies large portions of leaves as stems

For a single fold of the experimental evaluation, we selected one rosebush model for training and tested the algorithms on the remaining 10 models. For the unsupervised method based on local features on point clouds, we used the training model to optimize the weights of the data and smoothness terms. The results were averaged over the test models and over 5-fold experiments. A different rosebush model is reserved as training data for each fold. Table 4 gives the performances of the baseline leaf/stem segmentation methods. The visual results for a sample test rosebush are given in Fig. 2. The predicted labels of the rosebush model are displayed as a volume or as a point cloud depending on the type of the data the corresponding method processes. Figure 3 gives the stem points predicted by each baseline method. Correct predictions of the stem points with their connectivity maintained are especially important for establishing the architectural structure of the plant.

**Table 4:** Performances of the baseline leaf/stem segmentation methods (%)

Method	$R_{leaf}$	$R_{stem}$	$P_{leaf}$	$P_{stem}$	IoU$_{leaf}$	IoU$_{stem}$
LFPC-u	$95.74\pm 1.74$	$88.03\pm 1.82$	$98.23\pm 0.33$	$75.01\pm 9.76$	$94.10\pm 1.54$	$67.96\pm 8.18$
LFPC-s	$97.79\pm 0.46$	$80.50\pm 1.29$	$97.19\pm 0.48$	$83.67\pm 4.88$	$95.10\pm 0.46$	$69.57\pm 3.87$
LFVD	$\mathit{99.38 }\pm 0.13$	$90.01\pm 1.17$	$98.53\pm 0.43$	$\mathit{95.38 }\pm 1.02$	$\mathit{97.93 }\pm 0.47$	$\mathit{86.23 }\pm 0.31$
3D U-Net	$81.06\pm 1.68$	$\mathit{97.41 }\pm 1.43$	$\mathit{99.63 }\pm 0.29$	$54.0\pm 5.77$	$81.72\pm 1.71$	$53.58\pm 5.54$

**Fig. 4:**
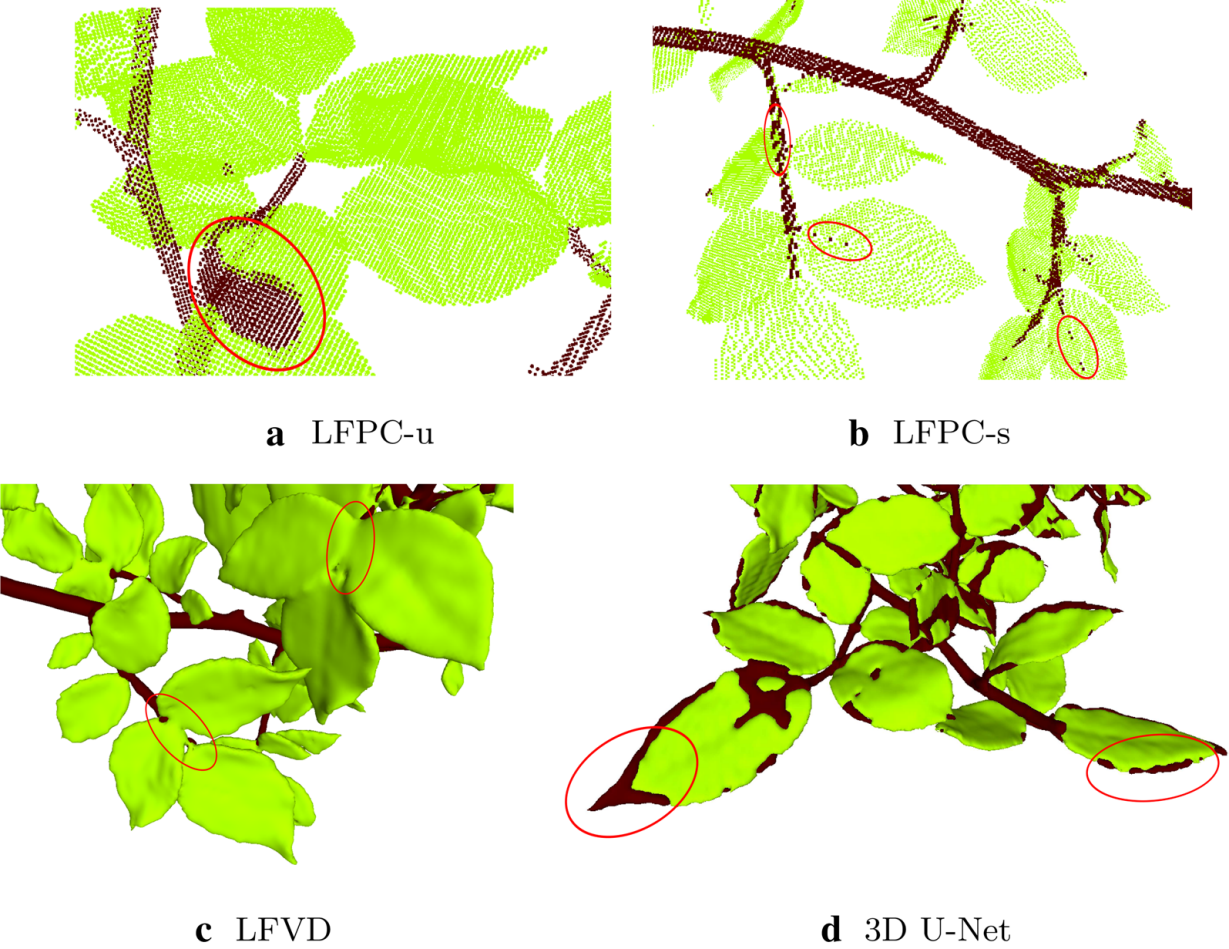
Examples to erroneous predictions of the baseline methods highlighted with red ellipses. The LFPC-u method (**a**) can classify an entire leaf or a portion of a leaf, especially at leaf borders with low curvature. With the LFPC-s method (**b**) we can observe isolated noisy predictions along the stem and on the leaves. Most of the errors occur at the midribs. The LFVD method (**c**) misclassifies the stem points on the petioles, which are in between close leaflets. The 3D U-Net (**d**) classifies boundaries and thick portions of leaves as stems

We can observe from Table 4 and Fig. 2 that the voxel classification method through local features (LFVD) gives the best overall performance for leaf/stem classification. It is a supervised method combining multi-scale volumetric local features with the random forest classifier. For this particular data set, it can model well the scale variations of leaf and stem points as well as their geometrical variations due to their locations on the organ (in the middle or at the border). The recall rate for the stem class is around 90%, meaning that 10% of the points on the branches are missed. Most missed stem points are on the petioles, which are in between close leaflets and possess an almost planar structure (Fig. 4c). The discontinuities in the stem-branch structure predicted by LFVD (Fig. 3c) generally correspond to the petiole portions just in between opposite leaflets.

The classification results obtained by LFPC-u are smooth (Fig. 2a) and the stem structure is mostly connected (Fig. 3a) due to the regularization imposed by the MRF formulation. However, smoothing labels of adjacent points in regions of low curvature leads to an entire leaf or a portion of it to be classified as stem if there is a smooth transition of normals at the boundary as seen in Fig. 4a. This propagation of labels through boundaries with low curvature causes a relatively low stem precision rate (Table 4). Likewise, smooth petiole and leaf boundaries lead to the classification of petiole points as leaves affecting the stem recall rate negatively. Although this method is unsupervised in the sense that it does not involve a classifier that learns feature transformations through labeled training data, the weights of the data and smoothness terms in Eq. 1 should be optimized for different plant species.

The performance of LFPC-s is slightly higher than that of LFPC-u in terms of the IoU metric (Table 4). Notice that we did not incorporate the MRF formulation for the baseline method LFPC-s, although it is completely applicable through setting the data term using SVM scores. Since no smoothness constraint is imposed on the labels, we can observe isolated noisy predictions along the stem and on the leaves (Fig. 2b). The predicted stem structure has unconnected small regions due to some leaf points classified as stems (Fig. 4b). Most of these errors occur at the midribs which are usually the thickest parts of the leaves.

**Fig. 5:**
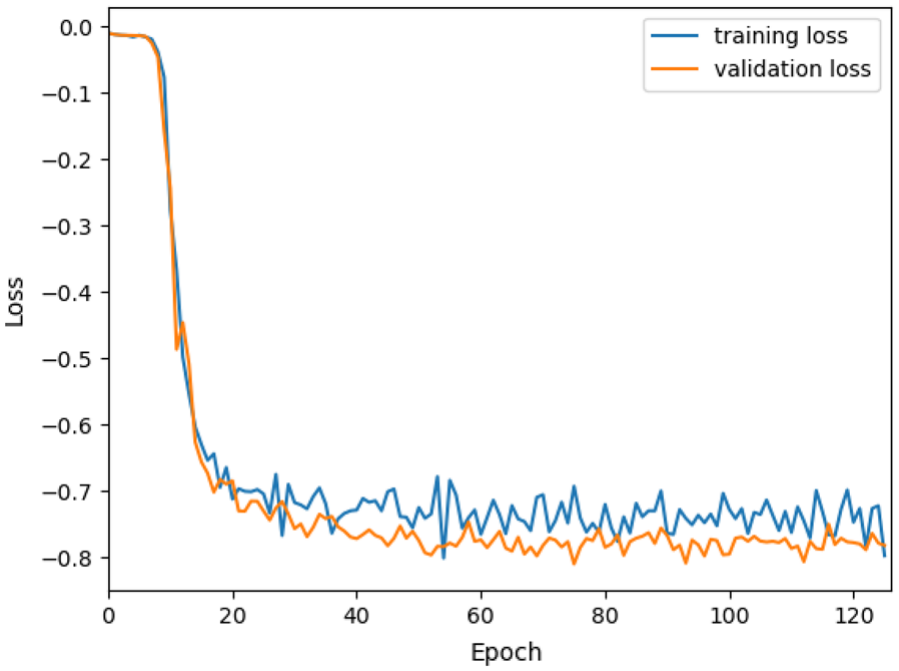
Evolution of loss for training and validation data with training epochs for 3D U-Net

3D U-net gives the lowest performance as compared to the other methods. Boundaries and thick portions of leaves are classified as stems as can be observed from Fig. 4d. We give in Fig. 5 the evolution of the training and validation loss. The dice coefficient function is used as the loss function in 3D U-Net algorithm, which shows a value in a range of 0 to 1. In this case, a negative is multiplied to values for optimization purposes. The curves in this figure show that the model can converge fast after about 50 epochs with the minimum overfitting between training and validation. However, the CNN network did not model the variations of leaves since we used sub-volumes from a single rosebush model for training to have a fair comparison with other baseline supervised methods. The 3D U-net has far more parameters to learn than the other methods; therefore, more training data is required for it to be properly trained. Besides, we directly applied the original 3D U-net architecture [47], which was designed for bio-medical data, without modification. In order to improve the results with deep learning, one can either increase the training data by using more than one rosebush model, employ data augmentation strategies, alter the 3D U-Net architecture or propose a new architecture suitable for 3D segmentation of plants. However, detailed analysis of the modifications on these lines is beyond the main objective of this work. We leave the design of 3D CNN architectures specific to plant organ segmentation as an open research problem, to the solution of which our entire labeled data set can contribute.

The methods LFPC-u, LFPC-s, and LFVD were run on a computer with an Intel processor of 3.5 GHz and 128 GB RAM. LFPC-u and LFPC-s were coded with MATLAB, while LFVD was implemented with Python. The average processing time for segmentation of a single model with LFPC-u is 4.2 min. The training time of the SVM classifier for LFPC-s is 5.1 min on average. The segmentation time for a test model with LFPC-s is 1.6 min. The training time of the Random Forest classifier for LFVD is 13.4 min, and the testing time is 3.3 min. The 3D U-Net was trained using Python on a computer with an Intel processor of 2.2GHz and 8 GPUs of 64 GB. The training time is 3 h, while segmentation time for a new test model is 4.3 min on average.

## Discussion

The ROSE-X data set includes high resolution 3D models of real rosebush plants, each of which was annotated at the voxel level with the corresponding botanical organ class. In this article, we focused on the step of segmentation of leaves and stems of automatic phenotyping pipelines. We provided a benchmark for proper comparison of current and future approaches for leaf/stem segmentation.

In this article, the focus has been on leaf segmentation from the stem. This is the first essential step in analyzing the shape and the architecture of the plant. Other questions can be addressed with the ROSE-X data set including issues raised by breeders, producers or consumers such as the study of interactions between genotype and environment on the one hand and phenotype and visual perception on the other. Such issues require the analysis of the growth and morphogenesis of the plant through effective phenotyping. With this objective in mind, it is possible to consider petiole segmentation, the distinction between leaflet and leaves, the detection of meristem along the stem, the analysis of the different part of the flower and the stage of development.

Also, the extraction and encoding of the architectural structure of the plant in the form of an organized collection of the main stem, second and higher order branches, and the branching locations is an important phenotyping task. Another task would be to extract geometrical characteristics of the individual architectural components and their spatial relationships, such as the length and width of the branch segments, petioles and their branching angles, leaf length, width, and area, together with the leaf inclination angles. These advanced botanical traits would be accessible with the spatial resolution of the 3D images of the proposed data set ROSE-X.

In order to evaluate the accuracy of phenotyping methods that aim to extract such more advanced botanical traits, we will release a forthcoming extension of the data set, with extended ground truth data in the form of geometrical properties of individual organs such as leaves, leaflets, petioles, stem segments, branching locations, and the spatial relationship between them.

We present the rosebush models in volumetric form, however, our main concern is to provide labeled data of plants with complex architecture for phenotyping methods that use the visible surface points of the plants as input. The conversion of the volumetric form to a point cloud via sampling or via ray casting from an arbitrary viewpoint is straightforward. As part of the future work, we will generate partial point clouds from the models as seen from around the plant, and apply phenotyping methods that rely on partial data.

Another important issue is the applicability of leaf/stem classification methods trained with the rosebush data set to other plant species. Future work will involve the expansion of the data set with 3D models of different species, and the adaptation of the classifiers learned from one species to others.

## Conclusion

This paper introduces a data set composed of 11 complete 3D models acquired through X-ray scanning of real rosebush plants. The models are stored in a voxel grid structure. We also provide the ground truth data, where each voxel stores the corresponding organ class label. The plant models are free from self-occlusion, however they posses complex architectural structure. As a sample application where the data set can be of use, we chose leaf-stem segmentation and compared the classification performances of four baseline methods. We observed that the volumetric approach (LFVD), where a random forest classifier is trained with local features, yielded the best performance. However, other baseline methods tested in this work are also open to further improvement, and there are yet the state-of-the-art techniques (Table 3) to be evaluated on our dataset. The data set is suitable to be annotated with more advanced traits and can be used as a benchmark for evaluation of automatic phenotyping methods that go beyond classifying plant points as leaves and stems.

## Supplementary information

**Additional file 1.** Description of the provided annotated dataset.

## Data Availability

Entire data set will be released after acceptance on a website of University of Angers. Only a single sample is made available in the submitted manuscript.
