# Supplementary material for: ROSE-X: an annotated data set for evaluation of 3D plant organ segmentation methods
Source: Plant Methods. 2020 Mar 4;16:28. doi: 10.1186/s13007-020-00573-w (PMC7057657; doi:10.1186/s13007-020-00573-w)
Supplement: Supplementary file 1 — Additional file 1. Description of the provided annotated dataset. [file 13007_2020_573_MOESM1_ESM.pdf]

ROSE-X: An annotated data set for evaluation of 3D plant organ segmentation methods
Helin Dutagaci; Pejman Rasti; Gilles Galopin; David Rousseau
Plant Methods

Additional file 1

Data formats of the 3D rosebush models

We provide the 11 models in the ROSE-X dataset in five different forms. The details about the file formats are as follows:

1) The raw X-ray image stacks, where the original X-ray images of the rosebushes are stored. The file format is 3D TIFF where multiple images are stored as a stack. The bit depth is 16. The list of the files is:

   S268650.tiff
   S268660.tiff
   S270230.tiff
   S270240.tiff
   S270250.tiff
   S271780.tiff
   S271790.tiff
   S271800.tiff
   S273080.tiff
   S273090.tiff
   S273110.tiff

2) A pair of image stacks for each model for the voxels of the shoot, the pot and the tag.
   The binary image stacks are provided as 3D TIFF files, with bit depth 1. The voxels with value 1 indicate the voxels on the shoot of the rosebush, the pot and the tag. The voxels with value 0 indicate the thresholded background voxels. The list of the files for the binary TIFF stacks is:

   S268650_binary.tiff
   S268660_binary.tiff
   S270230_binary.tiff
   S270240_binary.tiff
   S270250_binary.tiff
   S271780_binary.tiff
   S271790_binary.tiff
   S271800_binary.tiff
   S273080_binary.tiff
   S273090_binary.tiff
   S273110_binary.tiff

   The labels for the rosebush models are provided as 3D TIFF files, with bit depth 8. The values of the voxels correspond to the following labels:
   0 → Background
   1 → Leaf
   2 → Stem/branch
   3 → Flower
   4 → Pot
   5 → Tag
   The list of the files for the labels is:

   S268650_labels.tiff
   S268660_labels.tiff
   S270230_labels.tiff
   S270240_labels.tiff
   S270250_labels.tiff
   S271780_ labels.tiff

S271790_ labels.tiff
S271800_ labels.tiff
S273080_ labels.tiff
S273090_ labels.tiff
S273110_ labels.tiff

3) A pair of image stacks for each model for the voxels only on the surface of the plant shoot. The voxels that are not on the surface of the shoot are set to zero. The voxels corresponding to pot and the tag are also set to zero.
The binary image stacks are provided as 3D TIFF files, with bit depth 1. The voxels with value 1 indicate the voxels on the surface of the shoot of the rosebush. The rest of the voxels are set to zero. The list of the files for the binary TIFF stacks is:

S268650_shoot_surface_binary.tiff
S268660_shoot_surface_binary.tiff
S270230_shoot_surface_binary.tiff
S270240_shoot_surface_binary.tiff
S270250_shoot_surface_binary.tiff
S271780_shoot_surface_binary.tiff
S271790_shoot_surface_binary.tiff
S271800_shoot_surface_binary.tiff
S273080_shoot_surface_binary.tiff
S273090_shoot_surface_binary.tiff
S273110_shoot_surface_binary.tiff

The labels for the surface of the shoot are provided as 3D TIFF files, with bit depth 8. The values of the voxels correspond to the following labels:
0 → Background
1 → Leaf
2 → Stem/branch
The list of the files for the labels is:

S268650_shoot_surface_labels.tiff
S268660_shoot_surface_labels.tiff
S270230_shoot_surface_labels.tiff
S270240_shoot_surface_labels.tiff
S270250_shoot_surface_labels.tiff
S271780_shoot_surface_labels.tiff
S271790_shoot_surface_labels.tiff
S271800_shoot_surface_labels.tiff
S273080_shoot_surface_labels.tiff
S273090_shoot_surface_labels.tiff
S273110_shoot_surface_labels.tiff

4) A point cloud for each model for the points corresponding to the shoot, the pot and the tag. The point clouds are provided in PLY format. The color assigned to each point indicates the label of the point:
green [0 1 0] → Leaf
red [1 0 0] → Stem/branch
blue [0 0 1] → Flower
yellow [1 1 0] → Pot
cyan [0 1 1] → Tag

S268650_pointCloud_labels.ply
S268660_pointCloud_labels.ply
S270230_pointCloud_labels.ply
S270240_pointCloud_labels.ply
S270250_pointCloud_labels.ply
S271780_pointCloud_labels.ply

S271790_pointCloud_labels.ply
S271800_pointCloud_labels.ply
S273080_pointCloud_labels.ply
S273090_pointCloud_labels.ply
S273110_pointCloud_labels.ply

5) A point cloud for each model for the points corresponding to the points on the surface of the plant shoot.
The point clouds are provided in PLY format. The color assigned to each point indicates the label of the point:
green [0 1 0] → Leaf
red [1 0 0] → Stem/branch
blue [0 0 1] → Flower

S268650_pointCloud_shoot_surface_labels.ply
S268660_pointCloud_shoot_surface_labels.ply
S270230_pointCloud_shoot_surface_labels.ply
S270240_pointCloud_shoot_surface_labels.ply
S270250_pointCloud_shoot_surface_labels.ply
S271780_pointCloud_shoot_surface_labels.ply
S271790_pointCloud_shoot_surface_labels.ply
S271800_pointCloud_shoot_surface_labels.ply
S273080_pointCloud_shoot_surface_labels.ply
S273090_pointCloud_shoot_surface_labels.ply
S273110_pointCloud_shoot_surface_labels.ply

The 3D TIFF files can be read and visualized using ImageJ.

The PLY files can be visualized using MeshLab.
